# Combining structural MRI with Polygenic Risk Scores to disentangle unipolar and bipolar depression: a multimodal machine learning study

**DOI:** 10.1192/j.eurpsy.2025.286

**Published:** 2025-08-26

**Authors:** T. Cazzella, F. Colombo, M. Acconcia, L. Fortaner-Uyà, F. Calesella, B. Bravi, I. Bollettini, C. Monopoli, S. Poletti, F. Benedetti, B. Vai

**Affiliations:** 1 Psychiatry and Clinical Psychobiology Unit - Division of Neuroscience, IRCCS San Raffaele Hospital; 2 Division of Neuroscience, University Vita-Salute San Raffaele, Milan, Italy

## Abstract

**Introduction:**

The differential diagnosis between Major Depressive Disorder (MDD) and Bipolar Disorder (BD) heavily relies on clinical observation. However, the two disorders often show similar symptomatologic profiles, leading to high misdiagnosis rates. Reliable biomarkers are therefore crucial to accurately discriminate between MDD and BD and provide better treatments. In this regard, Machine Learning (ML) could represent a turning point in the field precision psychiatry, given its capability of making single-subject level predictions.

**Objectives:**

In the present work, we aimed at providing a biomarker-based differential diagnosis between MDD and BD. To that end, we implemented: *i)* a structural MRI-based ML model; ii) a combined ML model, trained on MRI data and Polygenic Risk Scores (PRS) for different psychiatric disorders.

**Methods:**

168 depressed patients (73 MDD, 95 BD) were recruited at the IRCCS San Raffaele Scientific Institute. All patients underwent T1-weighted and Diffusion Tensor Imaging scans. Voxel-Based Morphometry (VBM) measures were extracted with Computational Anatomy Toolbox 12 (CAT12). Fractional Anisotropy (FA), Axial Diffusivity (AD), Mean Diffusivity (MD), and Radial Diffusivity (RD) were extracted with Tract-Based Spatial Statistics (TBSS). PRS for MDD, BD, Schizophrenia, Attention Deficit/Hyperactivity Disorder, Anorexia Nervosa and Autism were computed for a subsample of 155 patients (67 MDD; 88 BD) through Infinium PsychArray 24 BeadChip. We trained a Multiple Kernel Learning (MKL) algorithm with voxel-wise VBM and DTI features, subsequently combining them with the extracted PRS.

**Results:**

The neuroimaging model achieved a Balanced Accuracy (BA) of 71.65% and an Area Under the Curve (AUC) of 0.77 (85.44% sensitivity, 57.86% specificity). All the features contributed to the prediction, with AD (63%) and MD (26%) as the most predictive. Adding PRS to neuroimaging resulted in an improved performance, reaching 74.18% BA and 0.77 AUC (90.97% sensitivity, 57.38% specificity). The most predictive features of the neuroimaging-PRS model were MD (56%) and AD (27%).

**Conclusions:**

Structural MRI discriminated between MDD and BD, and adding PRS to neuroimaging features improved the performance of the ML model. These results highlight the predictive power of structural neuroimaging for the differential diagnosis between MDD and BD, as well as prompting multimodal classifiers as a promising tool in the context of precision psychiatry.

**Disclosure of Interest:**

None Declared

